# A White Grape Juice Extract Reduces Fat Accumulation through the Modulation of Ghrelin and Leptin Expression in an In Vivo Model of Overfed Zebrafish

**DOI:** 10.3390/molecules26041119

**Published:** 2021-02-20

**Authors:** Giuseppe Montalbano, Alessandro Maugeri, Maria Cristina Guerrera, Natalizia Miceli, Michele Navarra, Davide Barreca, Santa Cirmi, Antonino Germanà

**Affiliations:** 1 Zebrafish Neuromorphology Lab, Department of Veterinary Sciences, University of Messina, 98168 Messina, Italy; gmontalbano@unime.it (G.M.); mcguerrera@unime.it (M.C.G.); agermana@unime.it (A.G.); 2 Department of Chemical, Biological, Pharmaceutical and Environmental Sciences, University of Messina, 98122 Messina, Italy; amaugeri@unime.it (A.M.); natalizia.miceli@unime.it (N.M.); mnavarra@unime.it (M.N.); davide.barreca@unime.it (D.B.); 3 Department of Pharmacy-Drug Sciences, University of Bari “Aldo Moro”, 70125 Bari, Italy

**Keywords:** polyphenols, flavonoids, zebrafish, diet-induced obesity, white grape juice, *Vitis vinifera*, lipolysis, *Danio rerio*

## Abstract

A caloric surplus and a sedentary lifestyle are undoubtedly known to be the leading causes of obesity. Natural products represent valuable allies to face this problematic issue. This study was planned to assess the effect of a white grape (*Vitis vinifera*) juice extract (WGJe) in diet-induced obese zebrafish (*Danio rerio*). Fish were divided into four different diet groups: (i) normally fed (NF); (ii) overfed (OF); (iii) WGJe-supplemented NF (5 mL/L in fish water); (iv) WGJe-supplemented OF. Body mass index (BMI) was extrapolated each week. After the fourth week, euthanized zebrafish were processed for both microscopic evaluations and gene expression analyses. OF zebrafish showed higher BMI values with respect to NF counterparts, an effect that was hindered by WGJe treatment. Moreover, histological analyses showed that the area of the adipose tissue, as well as the number, size, and density of adipocytes was significantly higher in OF fish. On the other hand, WGJe was able to avoid these outcomes both at the subcutaneous and visceral levels, albeit to different extents. At the gene level, WGJe restored the altered levels of ghrelin and leptin of OF fish both in gut and brain. Overall, our results support the anti-obesity property of WGJe, suggesting its potential role in weight management.

## 1. Introduction

Epidemiological evidence clearly shows that modern Western diet, together with sedentary lifestyle habits, represents the leading cause of obesity in industrialized and developing countries due to their high fat and simple carbohydrate content [1]. However, this pressing issue is not only confined to obesity itself, rather to the ailments derived from it. Noteworthy, chronic degeneration of organs and the impairment of their function caused by obesity are acknowledged to be a fertile soil for diseases affecting cardiovascular (i.e., atherosclerosis, coronaropathy, stroke, and hypertension) and metabolic (i.e., type 2 diabetes mellitus and hyperlipidemia) systems, along with much others [2]. Therefore, great efforts have been devoted to the search of remedies aimed at facing this issue. Apart from changing both diet and lifestyle or more drastic approaches like surgery, pharmacotherapy proved useful for this purpose, although a long path still needs to be walked before finding more valuable drugs with lesser side effects and more robust efficacy [3].

The plant kingdom offers a wide plethora of different classes of compounds that help us in counteracting the side effects of Western diet, among which polyphenols [4] and its major subcategory, flavonoids [5], stand out among all. Many plant species are known to be great sources of these polyvalent compounds, which have been already reported for their remarkable activities. Among these, citrus fruit (*Citrus* spp.) [6,7,8,9,10], olives (*Olea europaea*) [11,12], and grapes (*Vitis vinifera*), the three food pillars of the Mediterranean diet, are undoubtedly the most relevant ones.

Grapes, defined as either “white” or “red” depending on the content of anthocyanins of the outer peel, contain plenty of flavonols, flavanols as along with phenolic acids, compounds widely reported in the literature for their anti-inflammatory, antioxidant, anticancer, anti-infective activities, as well as their cardioprotective and neuroprotective effects [13,14,15]. Moreover, it has been also claimed that these properties of grape polyphenols jointly interact in the prevention of metabolic syndrome, a condition which is a cluster of other morbidities tightly related to obesity [16]. Despite the scientific community’s attention mainly focused on the red-skinned grape, we already highlighted the potentiality of a flavonoid-rich white grape juice extract (WGJe). In particular, we showed its capability in reducing the radiocontrast media-induced nephrotoxicity in a renal cell line [17], its antimicrobial activity and effect on biofilm production *in vitro* [18], its neuroprotective effects in a mouse model of multiple sclerosis [19], as well as its beneficial effect on mitochondrial activity in an *ex vivo* model of activated lymphocytes from human subjects [20]. On the basis of these data, the aim of our study was to evaluate the *in vivo* anti-obesity effect of WGJe in diet-induced obese (DIO) zebrafish, a widely employed model to faithfully mimic the human one.

## 2. Results

### 2.1. Chromatographic Analysis of WGJe

**Figure 1:**
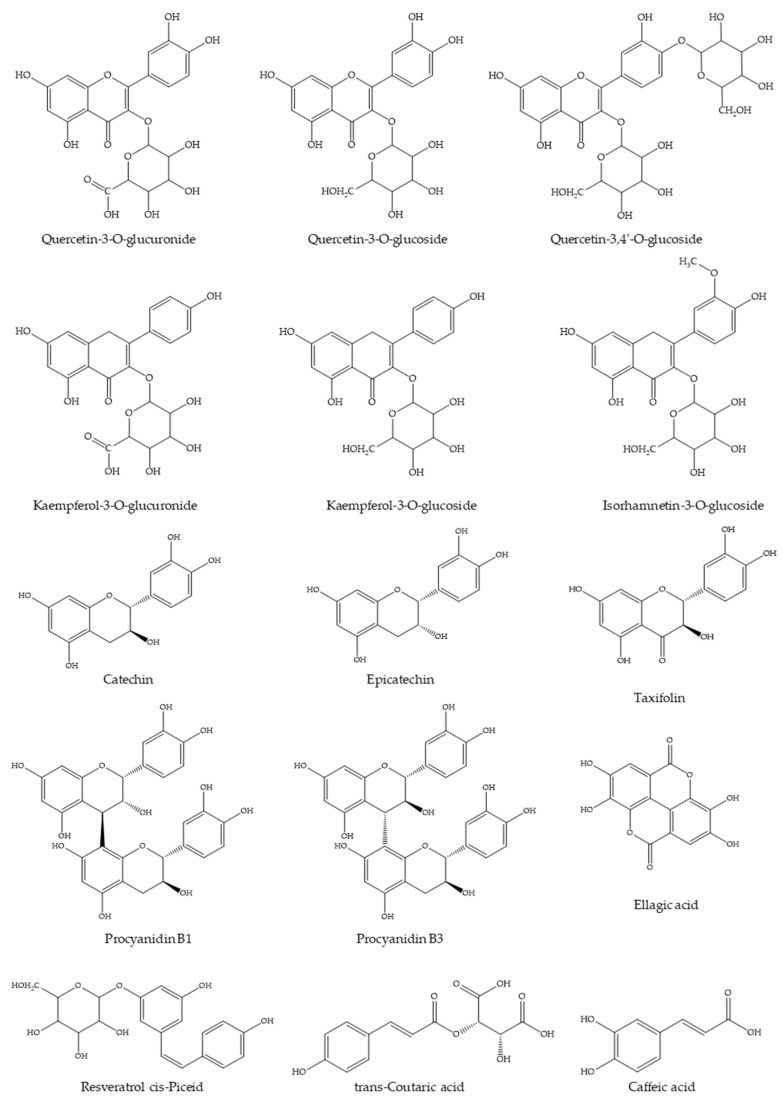
Chemical structures of the most abundant derivatives identified in WGJe.

The WGJe is particularly rich in phenolic and polyphenolic compounds, as can be observed from the data reported in Table 1 and in accordance with previously published data on the same natural matrix [17,19]. The most abundant compounds (ranging from ~300 to ~15,000 mg/kg) are quercetin, kaempferol, and isorhamnetin derivatives (as far as flavonols are concerned), procyanidin, catechin, and epicatechin (as far as flavanols are concerned), resveratrol (as far as stilbenes are concerned), ellagic acid, trans-coutaric acid, and caffeic acid (as far as phenolic acid are concerned), and taxifolin (as far as dihydroflavonols are concerned). The aglycones (such as quercetin and luteolin) have also been identified, although their total amount is clearly inferior to the corresponding glycosylated forms (Table 1). The chemical structures of the most representative compounds present in WGJe are depicted in Figure 1.

### 2.2. BMI Measurements

**Figure 2:**
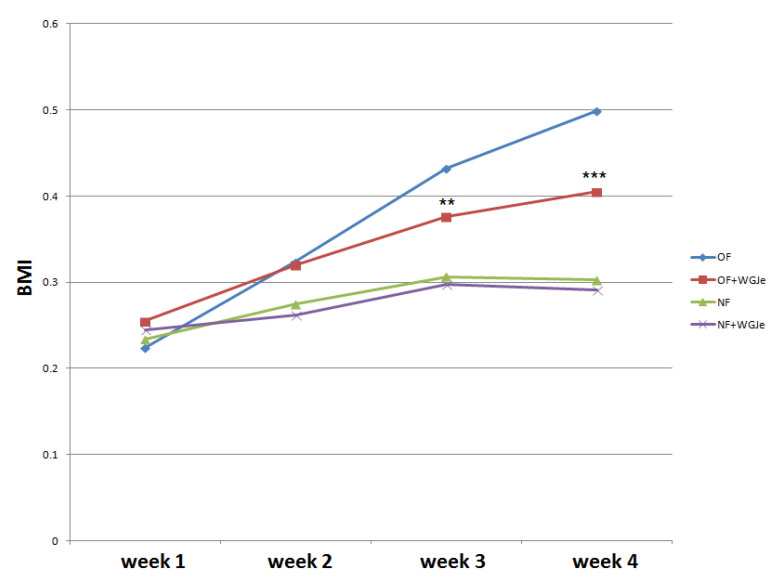
Effects of WGJe on body mass index (BMI) in overfed (OF) and normally fed (NF) zebrafish. The 4-week long experiment showed a constant growth of the BMI curve in OF group that decreased in the counterpart treated with WGJe. The difference between NF and NF + WGJe was not statistically significant. (** *p* < 0.01, *** *p* < 0.001, OF *vs.* OF + WGJe).

The experimental period lasted four weeks. Statistical analysis was performed to evaluate BMI variation among the four experimental groups, and a clear increase of BMI was observed in both treated (overfed (OF) + WGJe) and untreated OF groups with respect to normally fed (NF) ones, noticeable already after 2 weeks of treatment (Figure 2). However, the significant decrease of the BMI of the OF + WGJe group compared to that of the OF one between the third and fourth weeks of treatment (*p* < 0.01 and *p* < 0.001, respectively) indicated the specific role of WGJe supplementation in the control of metabolism. No statistically significant difference between treated and untreated NF groups was recorded during the experimental period (Figure 2).

### 2.3. Effect of WGJe on Adipose Tissue

**Figure 3:**
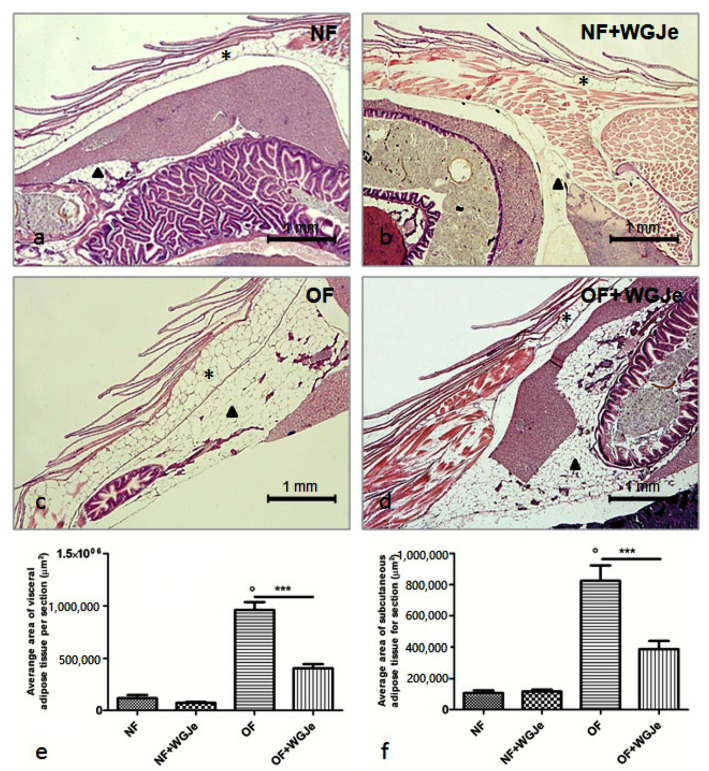
Histological micrographs of adipose tissue in the four experimental groups performed using hematoxylin and eosin staining (**a**–**d**). Morphometric analysis of visceral and subcutaneous total areas of adipose tissue (**e**,**f**). The fat deposit was poorly represented in both visceral and subcutaneous tissues of the NF group (**a**,**e**,**f**). Additionally, by the end of the experiment, the treated NF group did not show a significant change in fat deposit with respect to its untreated counterpart (**b**,**e**,**f**). The difference of the aforementioned tissues was statistically significant in the OF group with respect to the NF one (**c**,**e**,**f**). Similarly, the decrease at the end of the WGJe treatment in OF fish was statistically significant with respect to OF fish (**d**,**e**,**f**). Photos (**a**–**d**) are representative of those (three per section) taken in 10 fish per group. The values in graphs (**e**,**f**) are expressed in µm^2^ of adipose tissue per section. Scale bars are 1 mm. Subcutaneous adipose tissue is indicated with an asterisk, while visceral one with an arrowhead. Data presented in the graphs are the mean ± SD of 10 fish per group. *** *p* < 0.001 *vs.* respective counterpart; ° *p* < 0.001 *vs.* NF.

The morphological and morphometric analyses of adipose tissue after four weeks of treatment showed a differential growth and distribution of the aforementioned tissue among the different analyzed experimental groups (Figure 3). A statistically significant difference was found between NF and OF groups for both visceral and subcutaneous fat average area (*p* < 0.001), confirming that the obese model was correctly carried out (Figure 3a,c,e,f). A significant reduction of the visceral and subcutaneous adipose tissue area in the OF + WGJe *vs.* OF was detected (*p* < 0.001), highlighting the effect of WGJe (Figure 3c–f). However, WGJe did not produce any significant effect either at the visceral (Figure 3b,e) or at the subcutaneous (Figure 3b,f) level of NF fish.

**Figure 4:**
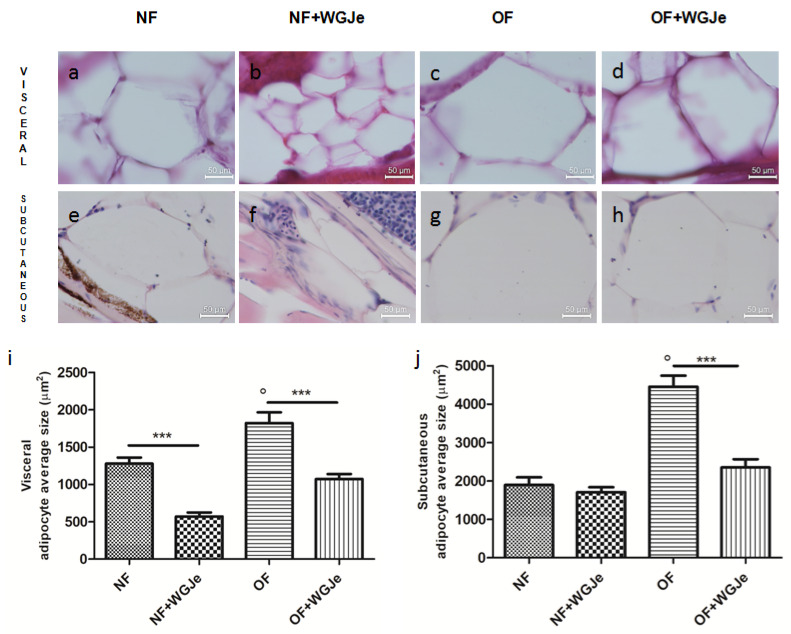
Outcomes of WGJe on visceral and subcutaneous adipocyte size. Histological confirmation (hematoxylin and eosin staining) of fat in the different experimental groups showing visceral (**a**–**d**,**i**) and subcutaneous (**e**–**h**,**j**) adipocyte size. The treatment with WGJe, particularly in OF group, reduced significantly in both visceral (**d**,**i**) and subcutaneous (**h**,**j**) tissues the average size of adipocytes. The photomicrographs (**a**–**h**) are representative of those (three per section) taken in 10 fish per group. The bar graphs show the results of the morphometric analysis of average adipocyte size in both visceral (**i**) and subcutaneous (**j**) tissue of the four experimental groups. Scale bars are 50 µm. Results from the morphometric analysis in graphs (**i**,**j**) are expressed in µm^2^ of adipocyte size and expressed as mean ± SD with *n* = 15 fish per group. *** *p* < 0.001 *vs.* respective counterparts; ° *p* < 0.001 *vs.* NF.

Differences in visceral and subcutaneous adipocytes size were observed among experimental groups. The adipocytes size in all the fat accumulation areas was significantly increased in the OF group *vs.* the NF group (*p* < 0.001; Figure 4a,c,e,g,i,j). Additionally, the treatment of WGJe in the OF group had halved (*p* < 0.001) the size of the subcutaneous and visceral adipocytes with respect to the OF alone group (Figure 4d,h,i,j). Statistically significant differences among the NF *vs.* the NF + WGJe groups were registered only in visceral adipose tissue (*p* < 0.001; Figure 4i).

**Figure 5:**
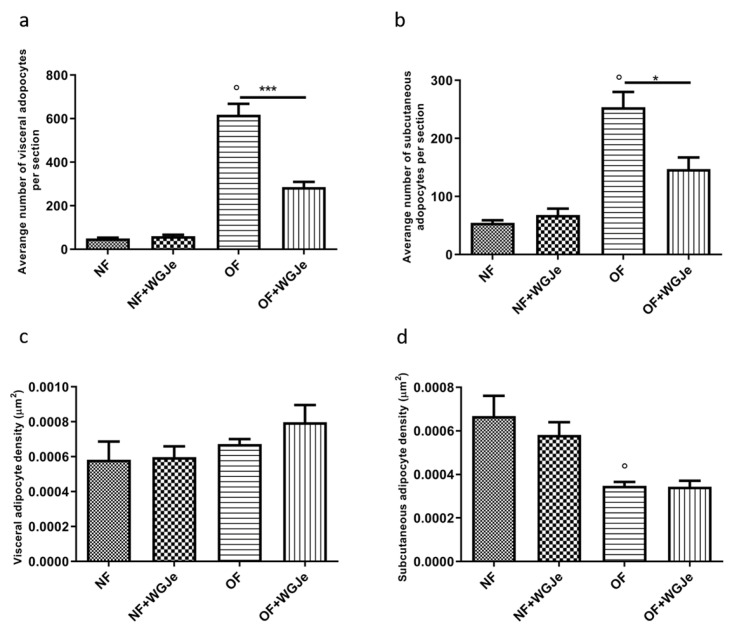
Variation of adipocyte cell number and density in WGJe-treated and untreated NF and OF zebrafish. The morphometric analysis of visceral (**a**) and subcutaneous (**b**) adipose tissue displayed a significant reduction of the number of adipocytes (**a**,**b**) in the OF + WGJe group compared to the OF untreated group. The adipocyte density was not statistically reduced at either visceral (**c**) or subcutaneous (**d**) levels. Values reported in the density graphs are expressed in µm^2^ of adipose tissue per section and represent the mean ± SD of 10 animals per group. * *p* < 0.05, *** *p* < 0.001 *vs.* respective counterparts; ° *p* < 0.001 *vs.* NF.

Another interesting aspect of the treatment effects with WGJe was evident in the OF + WGJe group in which a statistically significant reduction of visceral (*p* < 0.001) and subcutaneous (*p* < 0.05) adipocyte numbers were observed compared to that of the OF group (Figure 5a,b).

Furthermore, despite the higher visceral adipocyte density in the OF + WGJe group compared to the OF group, no significant difference was appreciated between both groups (Figure 5c). The density of subcutaneous adipocytes (Figure 5d) did not show any statistically significant difference between treated and untreated groups for both the NF and OF groups.

### 2.4. Effect of WGJe on Ghrelin and Leptin Expression in the Normal and Overfed Groups

**Figure 6:**
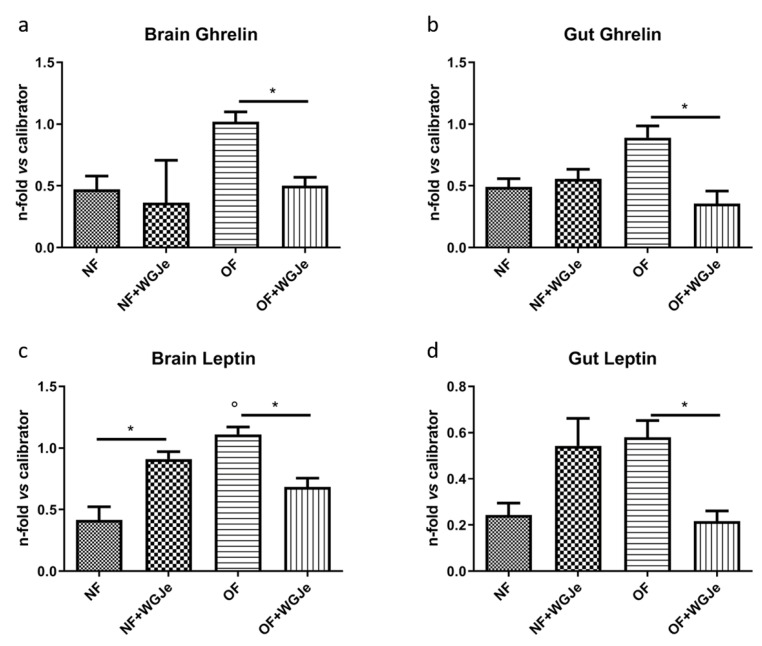
Effect of WGJe on the expression level of food-intake related genes. The expression of ghrelin (**a**,**b**) and leptin A (**c**,**d**) mRNA levels were investigated by qPCR analyses versus an arbitrary calibrator in both brain and gut of the normal fed (NF) and overfed (OF) groups, with and without WGJe treatment. Relative quantities of mRNA were calculated using the 2^−^^ΔΔCt^ quantification method. Results are expressed as fold change in WGJe-treated fish compared to those found in untreated ones after normalization to *β-actin*. Data represent the mean ± SD of five animals for each group. * *p* < 0.05 *vs.* respective counterparts; ° *p* < 0.01 *vs.* NF.

We analyzed the anti-obesity effects of WGJe on the expression levels of two hormones involved in food intake regulation: ghrelin, an appetite-stimulating hormone, and leptin, an anorexigenic one. Therefore, ghrelin and leptin mRNA levels were investigated by real-time PCR (qPCR) using an arbitrary calibrator in both the brain and gut of the NF and OF groups, with and without WGJe treatment (Figure 6). In the brain, ghrelin expression levels were higher in the OF compared to the control group (NF) (*p* < 0.05; Figure 6a). The administration of WGJe extract showed a significant reduction of ghrelin mRNA expression levels in the OF + WGJe group compared to the OF group (*p* < 0.05; Figure 6a). Similarly, WGJe extract was able to reduce ghrelin mRNA expression significantly in the gut of the OF + WGJe group compared to the untreated counterparts (OF) (*p* < 0.05; Figure 6b). The analysis carried out for leptin showed significantly higher levels in OF compared to the NF group in both brain and gut (*p* < 0.01 and *p* < 0.05, respectively; Figure 6c,d). The treatment with WGJe extract significantly decreased the leptin mRNA level in both the brain and gut of overfed (OF + WGJe) compared to the untreated group (OF) (*p* < 0.05; Figure 6c,d).

## 3. Discussion

Obesity represents one of the most crucial health problems in industrialized countries as well as a serious and emerging issue in developing ones. Obesity is due to an alteration of the strict relationship between caloric intake and energy expenditure, resulting in a positive energy balance. In overweight adults, the degree of obesity is classified using the body mass index and is often associated with serious comorbidities, such as metabolic disorders, type 2 diabetes mellitus, fatty liver disease, hypertension, myocardial infarction, stroke, as well as several cancers [21]. The strategy to contrast overweight is mainly based on lifestyle interventions, dietary modifications, physical activity and pharmacotherapy. In this view, the use of natural compounds instead of synthetic drugs could be a promising strategy to tackle weight gain and related comorbidities mainly, given the limited side effects [22]. Among them, WGJe, used in this study, is considered to be endowed with interesting health benefits. This extract is composed of several substances belonging to different chemical classes comprising flavonols, hydroxycinnamates, phenolic acids, and resveratrol with significant antioxidant and anti-inflammatory activities [13,19]. In this paper, we demonstrated for the first time the anti-obesity effect of WGJe in an experimental animal model of DIO zebrafish using an anatomical and molecular approach. It is well-known that the zebrafish is considered an intriguing and emergent experimental animal organism utilized as a model for human diseases. It has been well-demonstrated that the obesity in zebrafish induced with the diet shares many pathophysiological conditions with human one, such as the fat deposit localization and the involvement of the same orexigenic and anorexigenic genes [23,24,25]. Moreover, many functions related to food intake, appetite control, and lipid metabolism pathways were well preserved during evolution [26,27]. In this study, we used the well-consolidated model of DIO zebrafish daily supplemented with WGJe to analyze the effect of the diet supplementation on obesity, hyperplasia/hypertrophy of adipocytes, white adipose tissue storage, as well as gut and brain gene expression [23,28]. Obesity is always associated with chronic inflammation of white adipose tissue characterized by a dysregulated production of inflammatory cytokines (adipokines), including monocyte chemoattractant protein (MCP)-1, interleukin (IL)-8, IL-6, IL-1, tumor necrosis factor (TNF)-α, and anti-inflammatory IL-10, playing a key role in the onset of the obesity [29]. In our study, we demonstrate, first of all, that overfeeding the zebrafish for 6 weeks with 60 mg cysts/fish/day (20 mg cysts/fish three times a day) of freshly hatched *Artemia nauplii* allowed the development of a DIO zebrafish model with a remarkable increase of BMI and body fat accumulation at both subcutaneous and visceral level, in the liver, pancreas, and gut area. These data confirm that one of the principal features of obesity is represented by the increase in size and number of the adipose cells [30], also in zebrafish [27]. Then, the OF zebrafish group was compared with an obese zebrafish group supplemented daily with WGJe showing an evident reduction of the BMI and of the adipose tissue. Particularly, the decrease in size and in the number of adipocytes might suggest a potential role of WGJe in the metabolism of adipose tissue and in energy homeostasis, resulting in a lipolytic action and, hence, an anti-obesity effect [24,25,31,32]. This effect could also be explained by the well-known anti-inflammatory propriety of the bioactive compounds present in WGJe on white adipose tissue [13,33,34,35,36]. In this context, we recently reported the ability of this extract to hamper the inflammatory status also in an *in vivo* model of experimental autoimmune encephalomyelitis by targeting several markers like TNF-α and inducible nitric oxide synthase (iNOS), as well as nitrotyrosine, an acknowledged oxidative stress product [19]. Moreover, the antioxidant properties of WGJe have already been elucidated by us employing activated lymphocytes obtained from human subjects, where it hindered endogenous mitochondrial peroxide production and restored mitochondrial membrane potential, thus enhancing cellular redox defense [20].

It is well-known that natural products rich in polyphenols act on the expression of different appetite-controlling genes involved in the feeding behavior [37,38]. For this reason, we evaluated, using qPCR, the expression of the two most relevant orexigenic and anorexigenic genes, leptin and ghrelin, in both brain and gut of NF, OF, and OF + WGJe-treated zebrafish, and demonstrated that the mRNA levels were affected by WGJe treatment. Leptin is an anorexigenic hormone produced by fat cells and might be considered the hormone of satiety, exerting a pivotal role in food intake and appetite control [39]. The mRNA and plasma levels of leptin are low in the pre-prandial period and during fasting, becoming higher after food consumption [40]. In zebrafish, leptin is expressed and localized in a neuronal subpopulation of the hypothalamus arcuate nucleus as well as in the intestine entero-endocrine cells [31], acting as a regulator factor of food behavior between the brain and gut [41]. Zebrafish presents two leptin genes, leptin A and B, respectively. The former was examined for its close homology with human leptin and for the previously demonstrated expression in the liver and gut of zebrafish [42]. In this study, the down-regulation of leptin A in the OF + WGJe group, with respect to the OF one, was observed and might be ascribed to the reduction of the fat depots, consistently with previous data [43]. Moreover, in obesity, leptin levels are chronically higher, leading to the desensitization of its receptors, and hence a hampered response of this hormone [44]. Dietary polyphenols were shown to be valuable allies in restoring the sensitivity of cells towards leptin, thus reversing the hyperleptinemic status [45]. Therefore, we can hypothesize that WGJe may act as a “leptin sensitizer” in our experimental model, reducing leptin levels in treated OF zebrafish. Surprisingly, our extract was able to increase leptin levels in NF zebrafish at both brain and gut levels. This may be ascribed to the role of polyphenols present in grapes that were shown to slightly enhance leptin sensitivity, despite at serum level, only in non-obese animals that were kept in a particular photoperiod (i.e., light/dark cycle) [46]. The abovementioned experimental set-up was comparable to ours, and this hypothesis is also corroborated given the close relationship between serum levels of leptin and circadian rhythms. Ghrelin is a hormone produced by gastric fundus mucosa with an orexigenic effect stimulating the increase of food intake and body weight. Ghrelin level is high during starvation and pre-prandial period while it is lower after a meal. Moreover, it has been recently demonstrated, surprisingly for an anabolic peptide, that the ghrelin mRNA levels decrease during overfeeding and in obesity in a rodent model and in humans [47]. In this view, we found reduced ghrelin mRNA levels in the brain and in the gut of zebrafish with diet induced obesity, treated with WGJe with respect to an OF group, confirming the hypothesis of ghrelin resistance [43], also in zebrafish. Moreover, the decreased levels of ghrelin mRNA, together with the reduced number and size of adipocytes observed, might suggest the active involvement of ghrelin in adipocyte biology and lipogenesis [48], acting as adipogenic factors through the activation of the peroxisome proliferator-activated receptor-γ (PPARγ) and the cytosine-cytosine-adenosine-adenosine-thymidine (CCAAT)/enhancer-binding protein-α also in fish [49].

In the same experimental model employed in this study, we recently reported that a *Citrus sinensis* (L.) Osbeck (sweet orange) var. Tarocco extract (OJe) exerts a marked anti-obesity effect [32], reinforcing the relevance of flavonoids in this field. OJe is rich in flavanones like hesperidin which is known for its antiadipogenic and delipidating effects in both *in vitro* [50] and *in vivo* [51] models, along with sinensetin. The same compounds have also been identified in WGJe, where their potentiality is flanked by the remarkable presence of flavones, such as quercetin derivatives, flavanol, and stilbene compounds, whose involvement in the anti-obesity process has been recently reported [52]. A plethora of evidence shows the promising effect of quercetin as a potent antioxidant and anti-inflammatory agent against obesity pathogenesis [53,54]. Taken together, these observations provide a clearer picture of the potentiality of the WGJe employed in this study, shedding some light on the compounds responsible for the observed activities. However, it is not possible to exclude also synergistic and/or antagonistic effects due to the richness of compounds present in the phytocomplex, which can markedly modulate the overall activity of WGJe. In fact, although some compounds can have a direct effect on lipid metabolism, others (i.e., dihydrochalcones) can influence carbohydrate uptake [55] or inhibit the enzymes involved in the metabolization of polysaccharides into monomers [56], hence inducing caloric restriction with remarkable anti-obesity effects. In this frame, we [7,10,57] and others [58] have suggested that the combination of compounds present in phytocomplexes may synergistically act to convey a stronger response than the one achievable employing a single compound alone, highlighting the potential of natural matrices as a source of nutraceutical/drug with remarkable biological implications [59].

In conclusion, although obesity is a multifactorial complex disorder caused by a positive energy balance, some actions such as appropriate dietary behavior and the use of natural products could contrast the weight gain. Overall, our results demonstrated that WGJe exhibits a reduction of adipose tissue and an anti-obesity effect providing an interesting perspective for developing novel approaches against overweight and the related comorbidities using an innovative combination of different natural substances.

## 4. Materials and Methods

### 4.1. Ethics Statement

The experimental protocols employed in this study were in accordance with the principles outlined in the Declaration of Helsinki and with the Italian law regarding the care and use of laboratory animals (National Law n. 26/2014). The Italian Ministry of Health approved the experimental protocol (A.M. n. 50, 8 August 2013).

### 4.2. Drug

The WGJe we employed derived from white grapes (*Vitis vinifera*) juice provided by the company “Bono & Ditta” (Campobello di Mazzara, Trapani, Italy). The extraction method consisted of adsorbent resin-equipped columns that retained polyphenolic and dye compounds of the must-muted. Afterward, the elution was performed using 4% NaOH and then acidified. The final product was filtered, lyophilized, and kept at −20°C until reconstitution in phosphate-buffered saline prior to being used.

The phenolic and polyphenolic compounds present in WGJe were analyzed by UPLC/QqQ–MS/MS metabolite profiling utilizing a method already validated and reported in the literature for food analysis, including grapes [60], utilizing a Waters Acquity HSS T3 column 1.8 μm, 150 mm × 2.1 mm (Milford, MA, USA), kept at 40 °C with a flow of 0.4 mL/min. The injection volume was 2 μL. The molecules were thoroughly identified and quantified using orthogonal methods, i.e., the reference standard of each compound was employed to obtain the RT and the selection of multiple reaction monitoring (MRM) transitions of one or two qualifier ions, as reported in the reference work [60]. Each sample was tested three times and gave superimposable chromatograms. The results have been expressed as mean ± SD.

### 4.3. Zebrafish Husbandry and Management, Experimental Groups, BMI Measurements, and Tissue Preparation

In the present study, adult male zebrafish (*n* = 60, 12-months old), obtained from a breeding colony at C.I.S.S. (Centre of Experimental Ichthyopathology of Sicily, Department of Veterinary Science, University of Messina), were utilized and were kept at a constant temperature of 28.5 °C, fed once a day with a commercial feed. The experimental protocol involved the random division of zebrafish into four dietary groups (*n* = 15 fish per group): normally fed (NF), overfed (OF), NF supplemented with 5mL/L of WGJe in fish water (NF + WGJe), OF supplemented with 5mL/L of WGJe in fish water (OF + WGJe). The NF groups were fed once a day (7:00 am), while the OF groups were fed three times a day to get the obese model (7:00 am, 12:00 pm, and 5:00 pm). Zebrafish were separated into tanks with one zebrafish per 1 L, according to Montalbano and colleagues [24,25,32]. The NF group was fed with the equivalent of 20 mg cysts/fish/day of freshly hatched *Artemia* nauplii; the OF group was fed with the equivalent of 60 mg cysts/fish/day (20 mg cysts/fish three times a day) of freshly hatched *Artemia* nauplii; the NF + WGJe group was fed with the equivalent of 20 mg cysts/fish/day of freshly hatched Artemia nauplii and living in 5 mL of WGJe per liter of water; the OF + WGJe group was fed with the equivalent of 60 mg cysts/fish/day of freshly hatched Artemia nauplii and living in 5 mL of WGJe per liter of water. After feeding, we observed the fish until their dose was thoroughly eaten, as well as randomly checked them in order to assess any possible sign of change in behavior throughout the day. The water in all groups was changed every day. The entire protocol took four weeks to complete. Every week, both the body weight and the length of every fish were measured in order to evaluate the increase in BMI levels. At the end of the fourth week, the fish were fasted overnight and then euthanized with a lethal dose of 0.2 g/L of ethyl 3-aminobenzoate methanesulfonate (MS 222; Sigma, Saint Louis, MO, USA). Euthanized zebrafish were processed for both microscopic evaluations and qPCR analyses: brains and intestines from five fish per group were quickly removed and processed in order to isolate the RNA for qPCR. The remaining fish (*n* = 10 fish per group) were fixed in Bouin’s fixative and routinely processed for light microscopy.

### 4.4. Gene Expression of Ghrelin and Leptin

From whole zebrafish brains and guts, total RNA was extracted using the TRIzol reagent (Invitrogen, Carlsbad, CA, USA); 2 µg of total RNA was reverse transcribed into cDNA using the High-Capacity cDNA Archive Kit (Applied Biosystems, Foster City, CA, USA). The mRNA levels of ghrelin and leptin A were analyzed by qPCR using a TaqMan^®^ universal PCR Master Mix (Applied Biosystems) [25]. The primers used were designed on the mRNA sequences published for *ghrelin, leptin,* and *β-actin* as follows: *ghrelin* (GenBank accession no. EU908735.1): forward 5′-CAAGAGTGGGCAGAAGAGAA-3′, reverse 5′-CTGAAGCACGGGACCATATT-3′; *leptin A* (GenBank accession no. NM_001128576): forward 5′-CATCATCGTCAGAATCAGGG-3′, reverse 5′0-ATCTCGGCGTATCTGGTCAA-3′; *β-actin* (GenBank accession no. NM_131031): forward 5′-TTGCCCCGAGGCTCTCTT-3′, reverse 5′-AGTTGAAGGTGGTCTCGTGGAT-3′. The assays were performed in triplicate using a 7500 PCR real-time system (Applied Biosystems). The results were calculated through the 2^−ΔΔCt^ algorithm against *β-actin*, and expressed as the n-fold difference compared to an arbitrary calibrator, chosen as a higher value than ΔΔCts.

### 4.5. Morphological Fat Analysis

The morphological studies of zebrafish adipose tissue were carried out on histological sections following Montalbano and colleagues [24,25,32].

### 4.6. Statistical Analysis

The assays were carried out in triplicate. All experimental data are reported as mean ± SD. Statistical analyses of BMI values were performed by one-way repeated measures analysis of variance (ANOVA) and p-values lower than 0.05 were considered significant. Statistical analyses of gene expression and fat morphometry were performed by standard one-way ANOVA, and any difference was considered significant if *p* < 0.05 via the application of a Tukey’s test. For all statistical analyses, we employed GraphPAD software.

## Figures and Tables

**Table 1:** Identification and quantification of phenolic and polyphenolic compounds present in white grape juice extract (WGJe) analyzed by UPLC/QqQ–MS/MS, subdivided into the corresponding chemical classes and expressed as mean ± SD.

Classes	Compounds	R_t_	Concentration (mg/kg)
*Flavonols*	Quercetin-3-*O*-glucuronide	4.45	14,231 ± 1684
	Quercetin-3-*O*-glucoside	4.5	6308 ± 572
	Quercetin-3,4′-*O*-di glucoside	3.57	1227 ± 183
	Kaempferol-3-*O*-glucuronide	5.4	918 ± 75
	Kaempferol-3-*O*-glucoside	5.45	402 ± 67
	Isorhamnetin-3-*O*-glucoside	5.69	320 ± 46
	Quercetin-3-*O*-glucoside-arabinoside	3.9	97 ± 9
	Rutin	4.18	30 ± 2
	Quercetin	8.4	24 ± 3
	Kaempferol-3-*O*-rutinoside	5	2.0 ± 0.4
	Isorhamnetin-3-*O*-rutinoside	5.3	2.4 ± 0.3
*Flavanones*	Hesperidin	5.84	16 ± 3
*Flavanols*	Procyanidin B1	2.4	7682 ± 528
	Catechin	2.8	3157 ± 283
	Procyanidin B3	2.72	2210 ± 305
	Epicatechin	3.32	321 ± 42
*Stilbenes*	Resveratrol cis-Piceid	7.55	2003 ± 184
	Resveratrol trans-Piceid	6.56	53 ± 4
*Flavones*	Luteolin-7-*O*-glucoside	4.56	5.6 ± 0.7
	Luteolin	7.37	0.8 ± 0.1
*Polymethoxyflavones*	Sinensetin	9.84	0.4 ± 0.02
*Phenolic acids*	Ellagic acid	4.38	837 ± 52
	*p*-hydroxybenzoic acid	2.84	83 ± 0.7
	Vanillic acid	3.23	61 ± 5
	2,6-dihydroxy-benzoic acid	3.61	4.8 ± 0.7
	Methyl gallate	2.9	1.6 ± 0.2
	trans-coutaric acid	2.85	2853 ± 427
	Caffeic acid	3.19	322 ± 41
	Chlorogenic acid	2.76	30 ± 2
	*p*-Coumaric acid	4.04	21 ± 1.4
	Ferulic acid	4.52	17 ± 3
*Dihydroflavonols*	Taxifolin	4.72	451 ± 33
	Dihydrokaempferol	6.07	54 ± 2.6
*Dihydrochalcones*	Phloridzin	6.22	31 ± 2.4
	Trilobatin	6.72	14 ± 2

## Data Availability

The data presented in this study are available on request from the corresponding author.

## Sample Availability

Samples of the extract employed in this study are available from the authors.

## Abbreviations

NF	Normally fed
OF	Overfed
WGJe	White grape juice extract
